# Dnmt3a Protects Active Chromosome Domains against Cancer-Associated Hypomethylation

**DOI:** 10.1371/journal.pgen.1003146

**Published:** 2012-12-20

**Authors:** Günter Raddatz, Qing Gao, Sebastian Bender, Rudolf Jaenisch, Frank Lyko

**Affiliations:** 1 Division of Epigenetics, DKFZ-ZMBH Alliance, German Cancer Research Center, Heidelberg, Germany; 2 Whitehead Institute for Biomedical Research, Cambridge, Massachusetts, United States of America; Ludwig Institute for Cancer Research, University of California San Diego, United States of America

## Abstract

Changes in genomic DNA methylation patterns are generally assumed to play an important role in the etiology of human cancers. The Dnmt3a enzyme is required for the establishment of normal methylation patterns, and mutations in Dnmt3a have been described in leukemias. Deletion of Dnmt3a in a K-ras–dependent mouse lung cancer model has been shown to promote tumor progression, which suggested that the enzyme might suppress tumor development by stabilizing DNA methylation patterns. We have used whole-genome bisulfite sequencing to comprehensively characterize the methylomes from Dnmt3a wildtype and Dnmt3a-deficient mouse lung tumors. Our results show that profound global methylation changes can occur in K-ras–induced lung cancer. Dnmt3a wild-type tumors were characterized by large hypomethylated domains that correspond to nuclear lamina-associated domains. In contrast, Dnmt3a-deficient tumors showed a uniformly hypomethylated genome. Further data analysis revealed that Dnmt3a is required for efficient maintenance methylation of active chromosome domains and that Dnmt3a-deficient tumors show moderate levels of gene deregulation in these domains. In summary, our results uncover conserved features of cancer methylomes and define the role of Dnmt3a in maintaining DNA methylation patterns in cancer.

## Author Summary

Dnmt3a is generally assumed to be a de novo DNA methyltransferase that plays an important role in establishing DNA methylation patterns during embryogenesis. However, mutations in the human DNMT3A gene have been detected in various cancers, suggesting that the enzyme might also be relevant for DNA methylation in adult tissues and in tumors. We have established genome-wide methylation profiles at single base pair resolution to define Dnmt3a-dependent methylation changes in a mouse tumor model. Our results show that mouse tumors with a functional Dnmt3a enzyme are characterized by regional hypomethylation, while Dnmt3a-deficient tumors showed a uniformly hypomethylated genome. Further data analysis revealed that Dnmt3a is required for maintaining normal DNA methylation patterns specifically in gene bodies and in active chromosome domains. Our study thus defines the role of Dnmt3a in maintaining DNA methylation patterns and provides a paradigm for understanding the effects of DNMT3A mutations on human cancer methylomes.

## Introduction

Altered DNA methylation patterns have long been recognized as important hallmarks of human cancers [1]. Over the past 30 years, numerous studies have contributed towards establishing a general model of the cancer methylome and identified a global loss of methylation marks and the hypermethylation of promoter-associated CpG islands as their main features [2]–[4]. Two recent studies have used whole-genome bisulfite sequencing to provide important experimental confirmation for this model [5], [6]: Detailed comparisons between colon cancer and normal colonic mucosa methylomes revealed regions of focal hypermethylation, as well as long stretches of hypomethylated DNA (100 kb–5 Mb) covering half of the genome.

DNA methylation patterns are established and maintained by the three DNA methyltransferases Dnmt1, Dnmt3a and Dnmt3b [7]. These enzymes have repeatedly been implied in tumor formation, but their precise role in the generation of cancer-specific methylomes is far from understood. In mouse models, it has been shown that heterozygous mutations for Dnmt1 cause a global reduction in DNA methylation, which was associated with a reduced prevalence of intestinal tumors [8], whereas deletion of Dnmt3b led to reduced tumor size [9]. Consistent with its tumor promoting role, overexpression of Dnmt3b was shown to enhance intestinal tumor formation, which was accompanied by the emergence of methylation patterns that were similar to those usually observed in human colon cancers [10], [11]. However, genetic mutations in DNMT1 or DNMT3B have not been described in human tumors yet.

DNMT3A is traditionally considered as a de novo DNA methyltransferase that plays an important role in the establishment of methylation patterns during early embryogenesis [12]. Interestingly, recent studies have identified genetic mutations in the human DNMT3A gene in several hematologic malignancies, including acute myeloid leukemia, myelodysplastic syndrome and T-cell lymphoma [13]–[16]. Cancer-associated mutations of DNMT3A are associated with poor prognosis [13]–[15], which suggests that they might functionally contribute to the disease phenotype. However, the described methylation differences between DNMT3A wildtype and mutant patients appear limited [13], [14] and their significance for the disease phenotype has remained unclear. A more detailed methylation analysis was recently described in a mouse model where conditional deletion of Dnmt3a in hematopoietic stem cells caused pronounced differentiation defects in serial transplantation experiments [17]. Further analysis suggested that the absence of Dnmt3a caused both hypermethylation and hypomethylation of promoter regions, and deregulation of stem cell and differentiation genes [17].

Consistent with Dnmt3a acting as a tumor suppressor gene, we have shown that deletion of Dnmt3a promotes tumor progression in a mouse model for lung cancer [18], thus providing a defined experimental system to investigate Dnmt3a-dependent methylome changes in cancer. Immunoprecipitation of methylated DNA suggested that several thousand fragments were hypomethylated in Dnmt3a-deficient tumors. These fragments predominantly localized to intragenic regions, which indicated a preferential activity of Dnmt3a in gene bodies [18]. In an effort to comprehensively and quantitatively characterize Dnmt3a dependent methylation changes at single base-pair resolution we have now used whole-genome bisulfite sequencing to establish genome-wide methylation maps from normal lung tissue, from Dnmt3a wildtype tumors and from Dnmt3a knockout tumors. Our results uncover a novel function of this de novo methyltransferase in cancer, as Dnmt3a deficiency results in loss of methylation in large active chromatin domains.

## Results

### Sample selection and characterization

**Figure 1:**
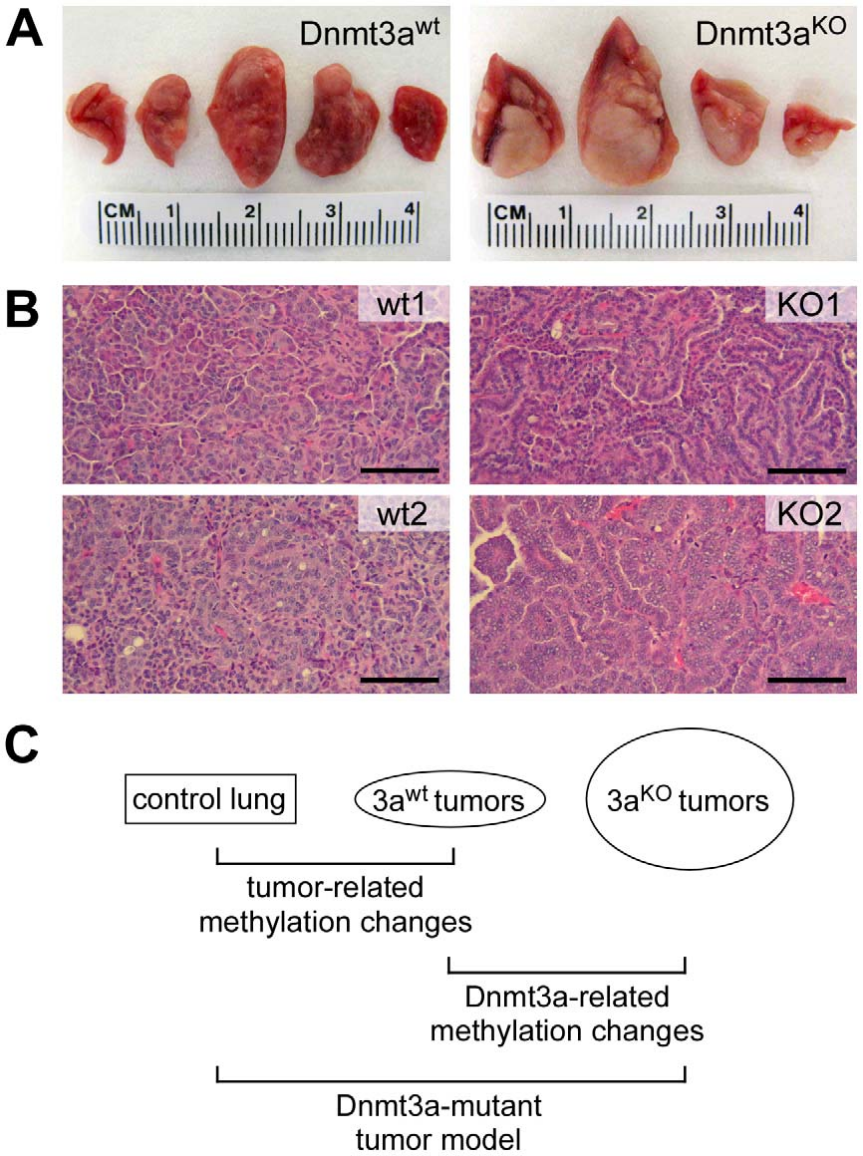
Overview of the experimental system used for this study. (A) Representative photographs of lungs from Dnmt3a wildtype and Dnmt3a deficient mice. Dnmt3a deficient mice have more advanced lung tumors. (B) Histophathology of two large Dnmt3a wildtype (wt) and two large Dnmt3a deficient tumors (KO). wt1: intermediate grade, solid growth pattern; wt2: intermediate to high grade, solid growth pattern; KO1: intermediate grade, papillary growth pattern; KO2: high grade, papillary growth pattern. Scale bars: 100 µM. (C) Schematic illustration of relevant comparisons for the characterization of methylation changes.

Our study is based on a mouse lung cancer model where tumor formation is initiated by conditional activation of an oncogenic K-ras allele [19]. Concomitant deletion of Dnmt3a in this model results in significantly more advanced tumors that are characterized by increased size, higher histological grade and papillary growth pattern [18] (Figure 1A and 1B). Notably, this experimental system relies on single, defined genetic mutations for tumor initiation and progression, and thus allows for the dissection of complex cancer-related DNA methylation changes (Figure 1C): (1) Tumor-related changes can be identified through comparisons between normal lung and Dnmt3a^wt^ tumors; (2) Dnmt3a-related changes can be identified through comparisons between Dnmt3a^wt^ and Dnmt3a^KO^ tumors; (3) methylation patterns of Dnmt3a mutant tumors can be modelled by identifying methylation differences between normal lung and Dnmt3a^KO^ tumors (Figure 1C).

In order to comprehensively characterize DNA methylation patterns in this model system, we used whole-genome bisulfite sequencing, which allows the generation of genome-wide methylation maps with single-base resolution [20], [21]. While Dnmt3a deficiency has been shown to increase the fraction of advanced tumors, this is accompanied by additional, more complex pathological changes [18]. To adequately reflect these complexities, we sequenced 5 libraries representing (1) normal lung tissue, (2) small and (3) large Dnmt3a^wt^ lung tumors, as well as (4) small and (5) large Dnmt3a^KO^ lung tumors. Sequencing libraries were generated as equimolar pools from 3–4 independent tissue samples (see Methods for details) to reduce the effects of stochastic epigenetic variation and to facilitate the identification of consistent epigenetic changes.

Paired-end sequencing on an Illumina HiSeq 2000 system with read-lengths of 105 bases generated 329 Gb of DNA sequence. After trimming to a maximal read length of 80 bases and a minimum base quality of a 30 Phred score, sequence reads were mapped to the MGSCv37 reference sequence using a mapping tool based on BSMAP 2.0. The resulting average genome coverage for individual samples ranged from 9.1× to 18.1×, with a combined genome coverage of 74× (Table 1).

**Table 1:** Sequencing data.

sample	number of reads (pairs)	mapping efficiency	coverage	Dnmt3a ratio	conversion rate
normal lung	176,609,911	81%	9.1×	0.90	98.17%
3a^wt^ (small)	410,418,317	60%	15.7×	1.40	98.76%
3a^wt^ (big)	326,342,429	69%	14.4×	0.77	99.22%
3a^KO^ (small)	368,253,086	77%	18.1×	0.15	98.85%
3a^KO^ (big)	347,746,475	77%	17.1×	0.05	98.94%

Coverage indicates the average genome coverage. Dnmt3a ratio indicates the coverage ratios of the targeted region of Dnmt3a (exons 17–19) and the complete Dnmt3a locus. Differences in the Dnmt3a ratio illustrate the different rates of Cre-mediated recombination. Bisulfite conversion rates were determined by analyzing the conversion of non-CpG dinucleotides (see Materials and Methods for details).

To confirm the presence of the tumor-inducing and -promoting genetic mutations, we analyzed the sequencing data for the K-ras and Dnmt3a loci. The results showed a slight reduction in the coverage of the Lox-Stop-Lox (LSL) cassette of the conditional LSL-G12D allele in control lung samples (Figure S1), consistent with the presence of 1 copy of the mutant allele in these heterozygous mice. In contrast, tumor samples showed a strong reduction in the coverage of the LSL cassette (Figure S1), which illustrates the efficient loopout of this element by Cre-mediated recombination. Examination of the sequencing data covering the Dnmt3a locus revealed no alterations in the coverage in the control lung and in the Dnmt3a wildtype samples (Figure S1). However, the coverage was strongly reduced in Dnmt3a deficient tumors and specifically in a region containing exons 17, 18 and 19 (Figure S1). This site-specific reduction in coverage reflects the Cre-mediated deletion of the targeted gene sequence. Notably, the deletion of this region appeared to be more efficient in large than in small knockout tumors (Table 1). For this reason, our subsequent data analysis was mainly based on comparisons between large tumors from Dnmt3a wildtype mice (Dnmt3a^wt^) and large Dnmt3a deficient tumors (Dnmt3a^KO^). It should be noted that we also included the sequencing data from small tumors in all steps of our analysis. Generally, the methylation patterns of large and small tumors of the same genotype appeared similar, indicating that the tumor size had no effect on the main results from our analysis (data not shown and see below).

### Overall methylation analysis

**Figure 2:**
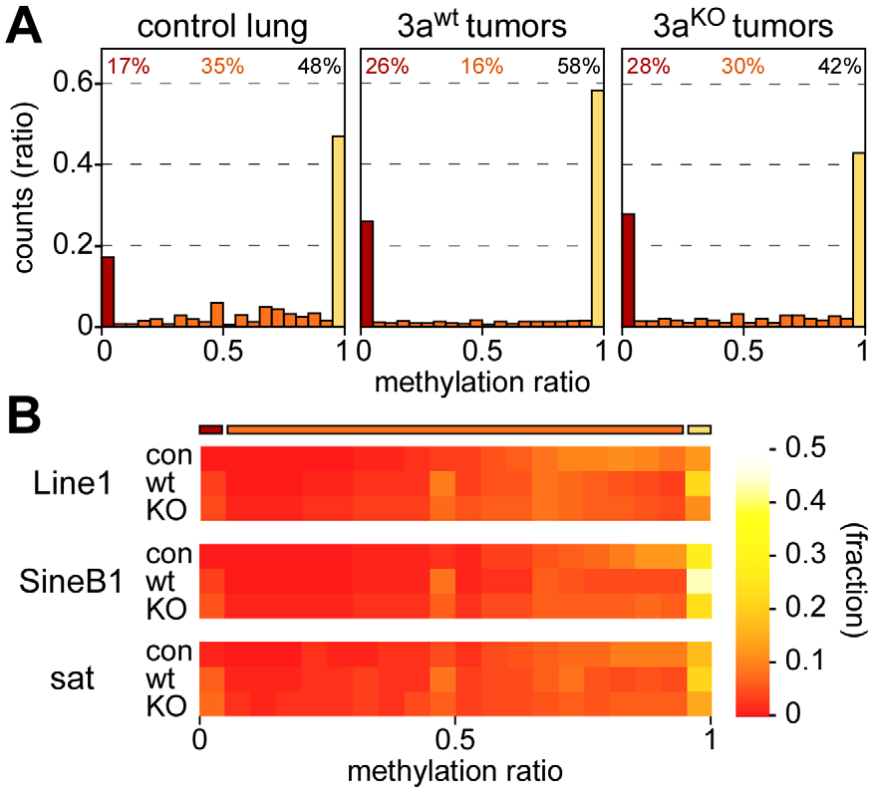
Methylation levels of CpGs and repetitive elements. (A) Methylation levels of individual CpGs in several tissue samples. Average methylation levels were determined for all covered CpG dinucleotides and then distributed into bins with increasing methylation ratios. Red bars bars indicate unmethylated CpG dinucleotides, orange bars partially methylated CpG dinucleotides and yellow bars completely methylated CpG dinucleotides. Percentages indicate the fractions of unmethylated (red), partially methylated (orange) and completely methylated (black) CpGs, respectively. (B) Color-coded histograms showing the distribution of repetitive element methylation in several tissue samples, as indicated. Average methylation levels were determined for all covered repeat elements and then distributed into bins with increasing methylation ratios.

As a first step towards a more detailed characterization of mouse lung tumor methylomes, we calculated the bisulfite conversion ratio for each library and found it to be >98% in all cases (Table 1). We then determined the methylation level for each of the 43 million CpG dinucleotides of the mouse genome. Average CpG methylation ratios were 0.68 in normal lung tissue, 0.66 in Dnmt3a^wt^ and 0.59 in Dnmt3a^KO^ lung tumors, thus suggesting a small but significant quantitative loss of DNA methylation in Dnmt3a^KO^ as compared to Dnmt3a^wt^ tumors. In further steps, all CpGs were sorted according to individual methylation ratios. This revealed a characteristic bimodal distribution in normal lung tissue, with a major fraction of CpG dinucleotides (48%) showing complete methylation, as indicated by a methylation ratio of >0.95. 17% of the CpGs were unmethylated (methylation ratio <0.05), while 35% of the CpG dinucleotides showed partial methylation ratios between 0.05 and 0.95 (Figure 2A). This distribution was clearly altered in Dnmt3a^wt^ lung tumors. The completely methylated and the unmethylated fractions increased to 58% and 26%, respectively, at the expense of the partially methylated fraction (decreased to 16%, Figure 2A). In Dnmt3a^KO^ tumors, the fraction of completely methylated CpGs became substantially reduced relative to Dnmt3a^wt^ tumors (42%, Figure 2A), while the fraction of partially methylated CpGs was correspondingly increased (30%, Figure 2A). These observations suggest a role of Dnmt3a in maintaining normal methylation levels for a subpopulation of genomic CpG dinucleotides. Very low levels of non-CpG methylation were also detectable in normal lung and appeared reduced in tumors (Figure S2). The fraction of partially methylated non-CpG dinucleotides was increased in Dnmt3a^KO^ tumors at the expense of fully methylated non-CpG dinucleotides (Figure S2). These findings are consistent with the role of Dnmt3a in non-CpG methylation [22].

Repetitive elements contain a large fraction of genomic DNA methylation marks (Table S1) and have been used as substitute markers for genomic DNA methylation changes. We therefore determined the methylation levels for all mapped copies of Line1, SineB1 and satellite repeat elements. The results showed that the majority of these repeats was highly methylated in normal lung tissue, with methylation ratios >0.5 (Figure 2B). This distribution was altered for all 3 elements in Dnmt3a^wt^ lung tumors, where both the fraction of completely methylated and the fraction of unmethylated sequences increased (Figure 2B). In Dnmt3a^KO^ lung tumors, the fraction of completely methylated elements was reduced relative to Dnmt3a^wt^ tumors, while the fraction of moderately to highly methylated repeats was increased (Figure 2B). Overall, the methylation changes at repetitive elements were very similar to those observed in our global analysis of CpG dinucleotides (Figure 2A) and thus further support the notion that Dnmt3a is required for effective maintenance methylation in a subset of sequences.

### Methylation of promoters, gene bodies, and intergenic regions

**Figure 3:**
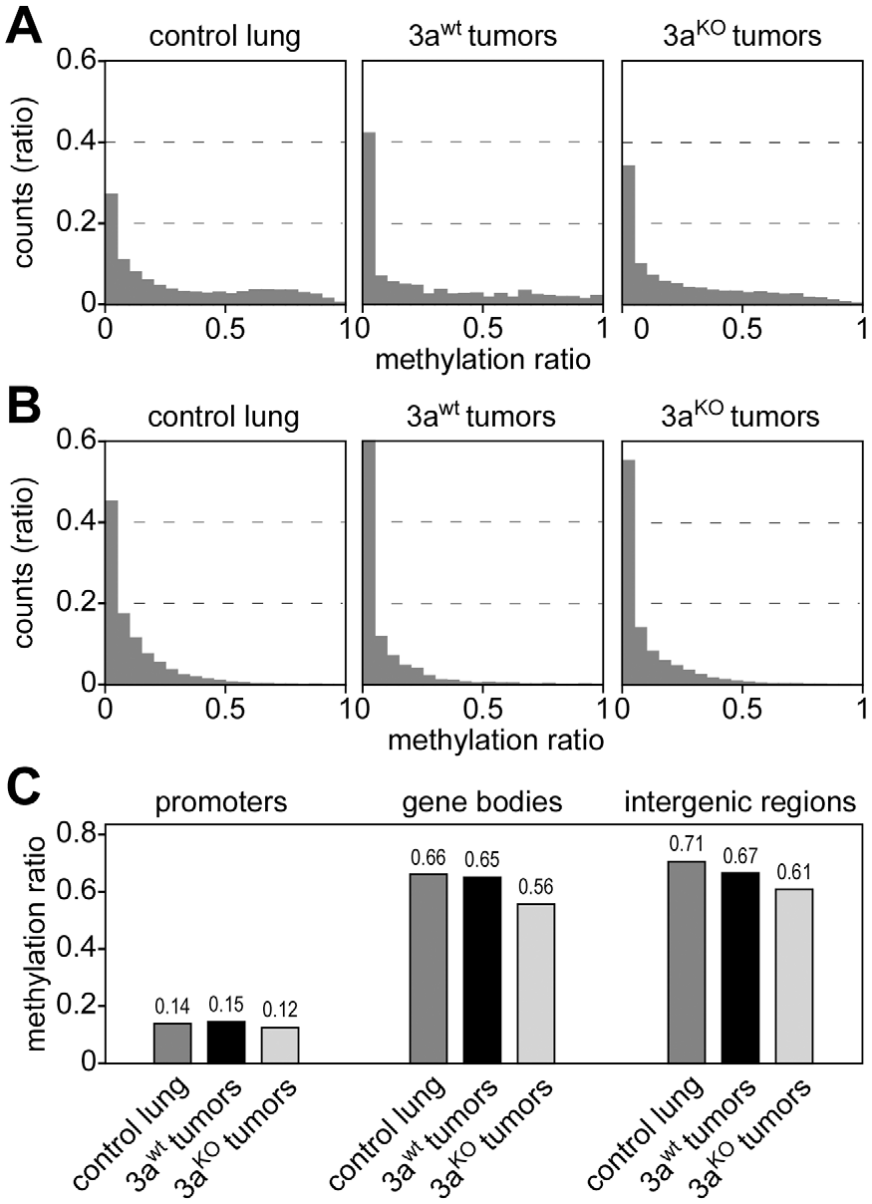
DNA methylation analysis of genetic subregions. (A) Histograms showing the distribution of promoter methylation in several tissue samples, as indicated. Average methylation levels were determined for all promoters (≥3 CpGs, coverage ≥3 reads) and then distributed into bins with increasing methylation ratios. (B) Histograms showing the distribution of CpG island-associated promoters. Average methylation levels were determined (>5 CpGs, coverage ≥3 reads) and then distributed into bins with increasing methylation ratios. (C) Average DNA methylation ratios of promoters, gene bodies and intergenic regions.

The observed unidirectional loss of methylation marks in Dnmt3a deficient tumors contrasts with a recent study that found both hypermethylation and hypomethylation at promoters in hematopoietic stem cells of Dnmt3a knockout mice [17]. We therefore defined promoters as 1-kb regions upstream of the transcriptional start sites of annotated genes and performed a detailed analysis of promoter methylation patterns in our dataset. Low levels of promoter methylation were seen in Dnmt3a^wt^ and Dnmt3a^KO^ samples (Figure 3A). The few promoters that were classified as hypermethylated were usually not conserved between large and small Dnmt3a^wt^ tumors (data not shown), which suggested that they were based on small quantitative differences and probably represent scoring artefacts. We also analyzed CpG island-associated promoters and again did not find any evidence for Dnmt3a-dependent promoter hypomethylation or hypermethylation (Figure 3B) in our system.

To investigate whether Dnmt3a deficiency differentially affects the methylation level of genetic subcomponents, we used the available genome annotation data to define promoter, gene body (5′-UTR, exon, intron and 3′-UTR) and intergenic regions. We then calculated average methylation levels for all three subregions in control lung, Dnmt3a^wt^ tumors and Dnmt3a^KO^ tumors. The results showed low levels for promoter methylation that did not change dramatically between genotypes (Figure 3C). The comparison between control lung and Dnmt3a^wt^ tumor indicated that hypomethylation in Dnmt3a^wt^ tumors preferentially occurred in intergenic regions (Figure 3C). In Dnmt3a^KO^ tumors, hypomethylation of intergenic regions became even more pronounced (Figure 3C). In addition, a distinct reduction in gene body methylation was observed in Dnmt3a^KO^ tumors, relative to both Dnmt3a^wt^ tumors and control lung (Figure 3C). These results confirm earlier findings [18] and also suggest a certain degree of complementarity between tumor-related and Dnmt3a-related methylation changes.

### Methylation changes in chromosomal domains

**Figure 4:**
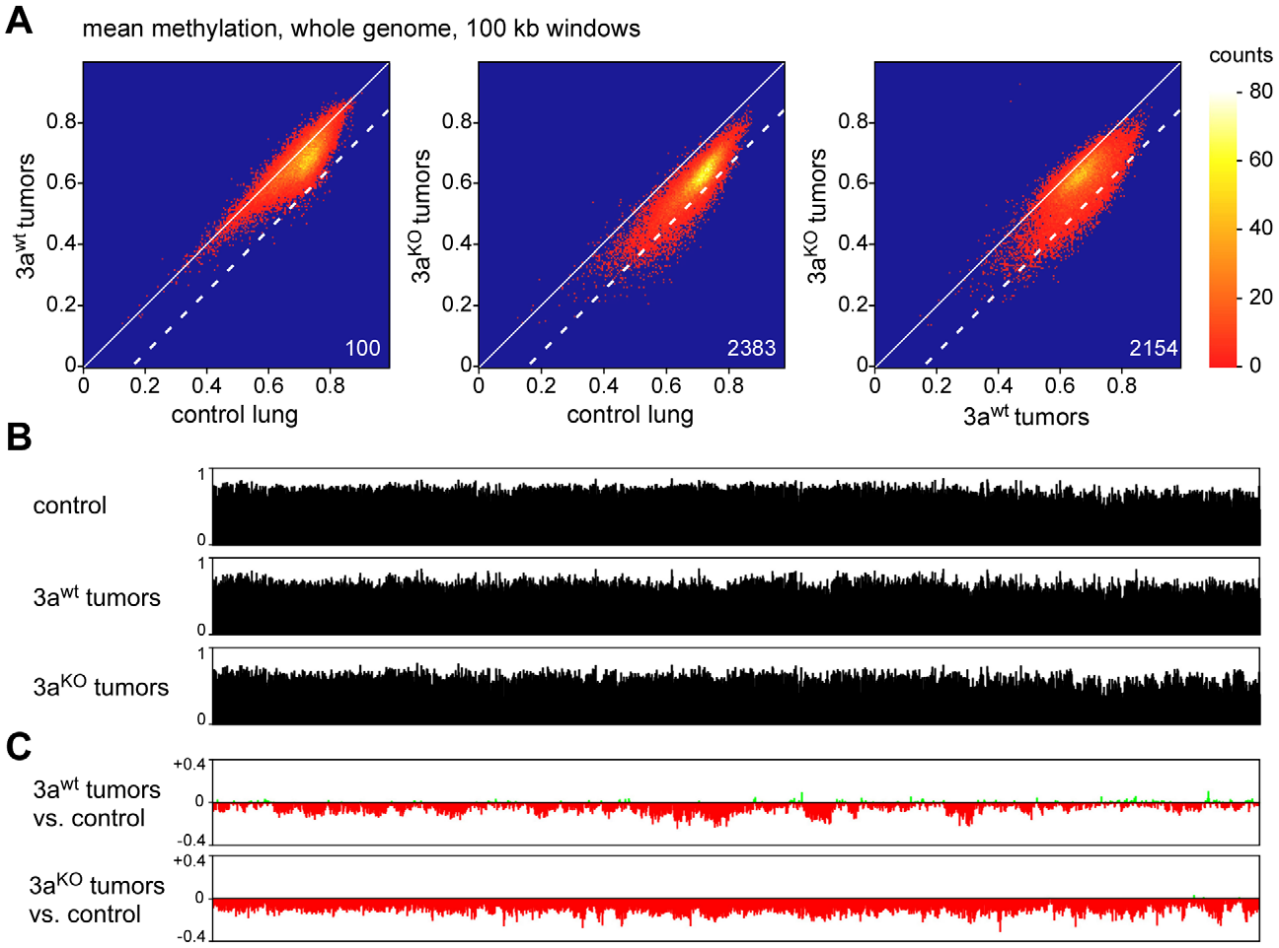
Analysis of large-scale DNA methylation patterns. (A) Density plots of average DNA ratios for 100-kb windows covering the entire mouse genome. Numbers indicate the number of windows with a methylation loss >0.15 (dotted line). (B) Methylation tracks consisting of 100-kb windows, covering the entire mouse chromosome 4. (C) Methylation changes of 100-kb windows covering the entire mouse chromosome 4. Methylation differences are plotted for Dnmt3a^wt^ lung tumors vs. normal lung and for Dnmt3a^KO^ lung tumors vs. normal lung, as indicated.

Recent studies have shown that human tumors are characterized by large partially methylated domains (PMDs) that extend over several 100 kb of DNA sequence [5], [6]. We therefore used a sliding window approach to identify similar hypomethylated areas in the mouse lung tumor samples. Various window sizes clearly identified hypomethylated domains across the entire genome (Figure S3; Figure 4A). More specifically, we counted 100 windows of 100 kb that were substantially hypomethylated (mean methylation ratio reduction >0.15) in Dnmt3a^wt^ tumors relative to control lung tissue. (Figure 4A). The number of hypomethylated windows was strongly increased when Dnmt3a^KO^ tumors were compared to normal lung tissue (n = 2383, Figure 4A), which indicated a pronounced effect of the Dnmt3a mutation on the cancer epigenome. This was further confirmed by a direct comparison of the methylomes from Dnmt3a^wt^ and Dnmt3a^KO^ tumors, which identified 2154 hypomethylated windows (Figure 4A). Similar effects were observed with various cutoffs for hypomethylation (Table S2), which suggests that the Dnmt3a mutation promotes the formation of large hypomethylated domains.

To characterize the changes in genomic methylation patterns in greater detail, we generated chromosomal methylation tracks by plotting average methylation levels of 100 kb windows for entire chromosomes (Figure 4B). A comparison between control lung and Dnmt3a^wt^ tumors further confirmed the presence of tumor-specific PMDs, which ranged from several 100 kb to several Mb of DNA sequence (Figure 4C). Strikingly, this domain structure was strongly altered in Dnmt3a^KO^ tumors and chromosomes were characterized by uniform hypomethylation (Figure 4C) suggesting that Dnmt3a may protect the genome from global cancer-associated hypomethylation.

**Figure 5:**
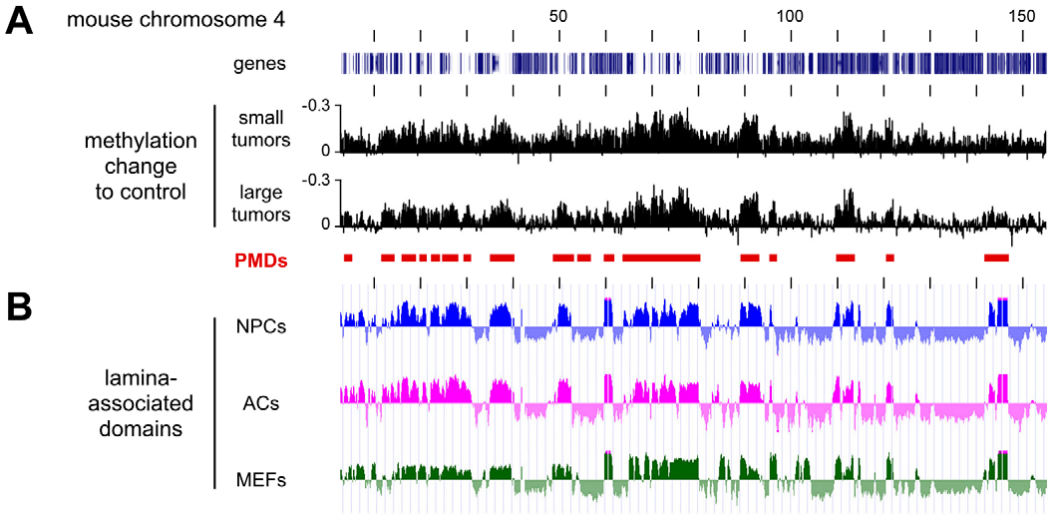
Characterization of hypomethylated domains in Dnmt3a^wt^ tumors. All tracks show data for the entire mouse chromosome 4, the distance between the vertical black lines corresponds to 10 Mb of DNA sequence. (A) Methylation differences between two independent sets of Dnmt3a^wt^ tumors (small and large) and normal lung. Conserved partially methylated domains (PMDs) are indicated as red bars. (B) PMDs coincide with lamina-associated domains (LADs). Lamin B1 binding profiles are shown for mouse neural precursors (NPCs, blue), astrocytes (ACs, magenta), and embryonic fibroblasts (MEFs, green).

For a more detailed analysis of PMDs, we also calculated the methylation differences between normal lung and the second set of Dnmt3a^wt^ tumor samples (small tumors, Figure 5A). This revealed that overall hypomethylation patterns (relative to normal lung) and the position of the PMDs were highly similar in the two sets of tumor samples (Figure 5A). Two previous reports had indicated that in human colorectal tumors PMDs correspond with lamina-associated domains [5], [6]. Lamina-associated domains (LADs) range in size from 100 kb to several Mb and represent major structural elements in genome organization [23]. LADs have also been mapped in mouse cells and showed only relatively minor variations between cell types [24], which suggests that their localization is conserved between tissues. When we correlated our methylation data from Dnmt3a^wt^ lung tumors to the available lamina interaction data [24] we observed a strong correspondence between PMDs and LADs (Figure 5B). These results strongly suggest that an important feature of human cancer methylomes is conserved in mouse tumors.

### Characterization of Dnmt3a-protected domains

**Figure 6:**
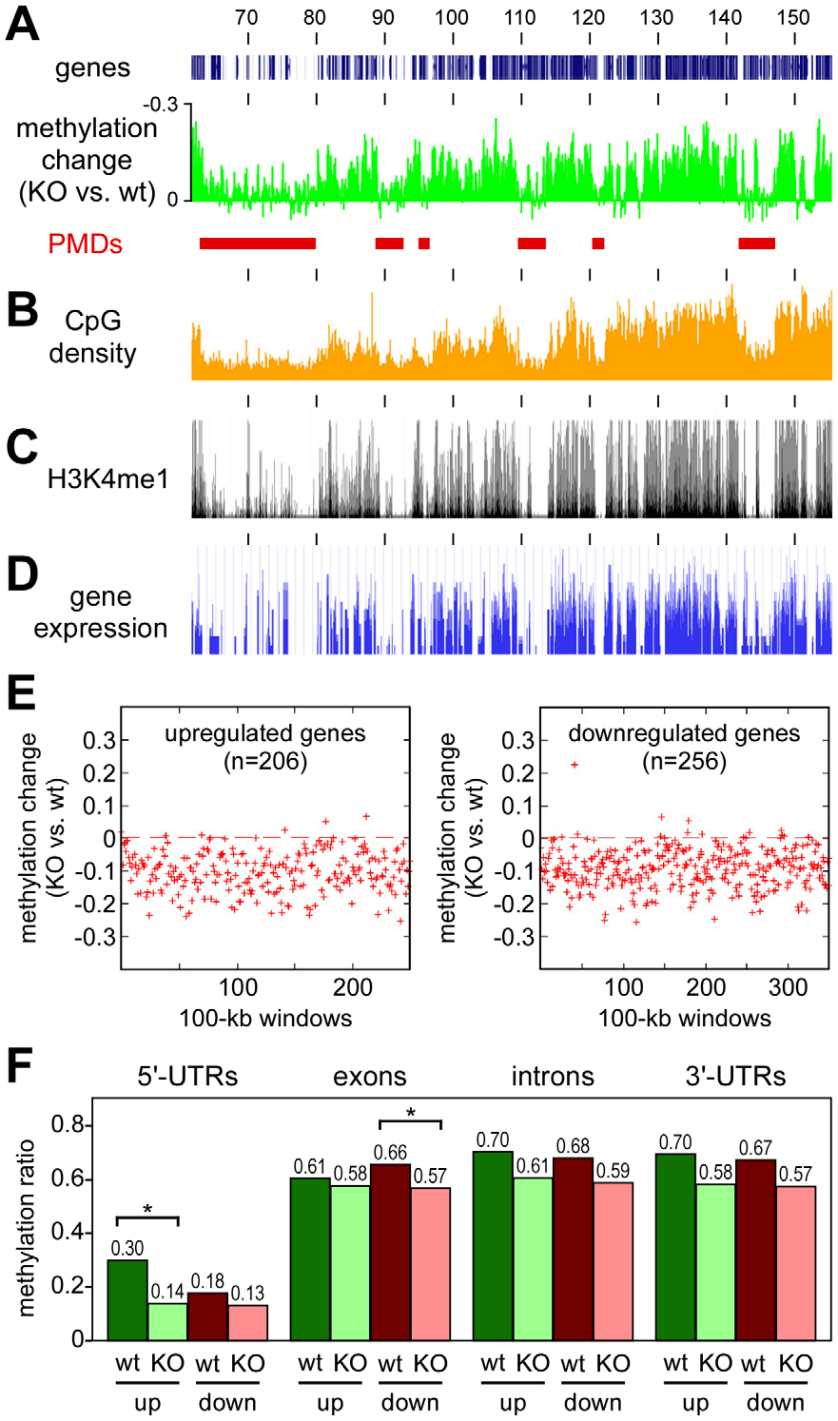
Characterization of Dnmt3a-protected domains. All tracks show data for the distal part of mouse chromosome 4, numbers correspond to chromosomal positions in Mb. (A) Methylation differences between Dnmt3a^KO^ and Dnmt3a^wt^ tumors. Peaks represent DPDs (red bars). (B) CpG densities plotted in 100 kb windows. (C) Distribution of H3K4me1 in normal mouse lung tissue. (D) Gene expression in normal mouse lung tissue. (E) Deregulated genes are associated with DPDs. Comparative gene expression profiling [18] identified 206 genes with >2-fold increased and 256 genes with >2-fold decreased transcript levels between in Dnmt3a^KO^ tumors (relative to Dnmt3a^wt^ tumors). Red marks show the methylation differences of gene-associated 100-kb windows in Dnmt3a^KO^ and Dnmt3a^wt^ tumors. (F) DNA methylation analysis of genes with Dnmt3a-dependent expression changes. DNA methylation patterns were analyzed for genes that showed >2-fold increased (up) or decreased (down) transcript levels between Dnmt3a^wt^ (wt) and Dnmt3a^KO^ (KO) tumors. Bars show average methylation ratios, calculated over all the gene regions being considered in each set. Differential methylation changes between upregulated and downregulated genes were detectable in 5′-UTRs and in exons. Within these regions, methylation differences between Dnmt3a^wt^ and Dnmt3a^KO^ tumors were statistically significant (P<0.01, as determined by a t-test) for 5′-UTRs of upregulated genes and for exons of downregulated genes. Methylation differences for 5′-UTRs of downregulated genes and for exons of upregulated genes were not statistically significant (P>0.01, as determined by a t-test).

Finally, we also sought to characterize the domains that retain normal methylation in Dnmt3a^wt^ tumors but become hypomethylated in Dnmt3a^KO^ tumors. A direct comparison of the methylation patterns between Dnmt3a^wt^ and Dnmt3a^KO^ tumors confirmed that the methylation loss associated with the Dnmt3a mutation appeared to be enriched in distinct domains (Figure 6A), which we termed Dnmt3a-protected domains (DPDs). Of note, DPDs showed a highly complementary distribution to PMDs (Figure 6A) and also a strong negative correlation with features that were associated with PMDs. This is exemplified by the colocalization of DPDs with regions of higher CpG density (Figure 6B) and a robust and highly significant negative correlation between CpG density and PMDs (r = −0.64, P<2.2×10^−16^). We also analyzed the distribution pattern of H3K4me1, a cell type-specific marker of active regulatory regions [25], in normal mouse lung tissue. The results showed a notable co-localization of H3K4me1 and DPDs (Figure 6B), while PMDs were depleted for H3K4me1 (r = −0.70, P<2.2×10^−16^). A similar pattern was also observed when gene expression profiles of normal mouse lung were analyzed (Figure 6C). This colocalization of DPDs with domains of active gene expression suggests that Dnmt3a deficiency might affect gene expression levels.

To determine the effect of the Dnmt3a-dependent methylation changes on gene expression, we re-analyzed the available gene expression data for this experimental system [18] using more stringent criteria for the identification of differentially expressed genes. This identified 462 genes that showed a more than 2-fold difference in steady-state transcript abundance between Dnmt3a^wt^ and Dnmt3a^KO^ tumors. In agreement with earlier observations [18], we observed bidirectional changes with 206 genes showing higher transcript levels in Dnmt3a^KO^ tumors and 256 genes showing lower transcript levels. Both groups of genes revealed a significant enrichment for biological terms that were predominantly associated with cell communication and adhesion (Table S3), which can be interpreted to reflect the more aggressive growth pattern of Dnmt3a-deficient tumors [18]. Of note, the vast majority of the up- and downregulated genes was found in regions with Dnmt3a-dependent hypomethylation (Figure 6E), suggesting that gene body hypomethylation in Dnmt3a-deficient tumors can have differential effects on gene expression. To further investigate this association, we analyzed DNA methylation patterns within gene bodies. Interestingly, this revealed differential methylation changes in up- and downregulated genes (Figure 6F). While upregulated genes were characterized by hypomethylation of relatively highly methylated 5′-UTR regions, downregulated genes showed a more pronounced hypomethylation of relatively highly methylated exons (Figure 6F). No differential effects were observed in introns and 3′-UTRs, which had similar levels of Dnmt3a-dependent hypomethylation for up- and downregulated genes (Figure 6F). These findings establish a close association between Dnmt3a-dependent DNA methylation and gene regulation. Further work will be required to understand the molecular function of Dnmt3a-mediated gene body methylation in the regulation of gene expression.

## Discussion

To date, DNMT3A represents the only DNA methyltransferase gene that has been found to carry genetic mutations in human cancers. The association of DNMT3A mutations with poor prognosis [13]–[15] and the functional effects of DNMT3A mutations on human cells and mouse tumor models [14], [18] suggest that the loss of the enzymatic activity can drive tumorigenesis by inducing tumor-promoting epigenetic lesions. However, human tumors are characterized by a complex pattern of genetic mutations and the specific effects of DNMT3A mutations on the cancer methylome have yet to be identified.

In our mouse model, lung tumor formation is initiated by a specific oncogenic mutation, i.e. the activation of the oncogenic K-ras allele in single cells. Dnmt3a is expressed in normal mouse lung tissue and in K-ras dependent mouse lung tumors, and the deletion of Dnmt3a has been shown to promote K-ras dependent lung tumor progression [18]. As such, this system represents a unique model to investigate the cancer-associated methylation changes and to identify the epigenetic alterations that underpin the tumor-promoting effects of Dnmt3a mutations.

A previous report provided evidence that conditional deletion of Dnmt3a in hematopoietic stem cells results in both hyper- and hypomethylation of promoters [17]. The observed gain of methylation marks appears difficult to reconcile with the loss of DNA methyltransferase activity, which raises the possibility that the observed methylation differences may be a consequence of the impaired differentiation in Dnmt3a-deficient hematopoietic stem cells. Indeed, our data show an unambiguous loss of methylation marks in Dnmt3a mutant tumors, as expected from a DNA methyltransferase-deficient model.

Large domains with reduced levels of DNA methylation have been described as a major feature of human colon cancer methylomes [5], [6]. Our data show that a single oncogenic mutation (a constitutively active mutant K-Ras allele) is sufficient to induce similar hypomethylated domains in mouse lung tumors. While the separation between normally methylated and hypomethylated domains appeared to be less clear than for the human cancer methylomes, this may be related to the experimental design of our analysis that was based on pooled samples to minimize inter-individual variation. Importantly, the hypomethylated domains identified in the tumor samples showed a strong correspondence with lamina-associated domains, which confirms previous observations from human colon cancers [5], [6] and suggests that partially methylated domains (PMDs) represent a conserved feature of tumor methylomes. PMDs are gene-poor, inactive domains and coincide with structural elements in the organization of the genome (lamina-associated domains) [23]. It is possible that PMDs represent genomic regions that have lower functional requirements for faithful methylation maintenance and that maintenance methylation in these domains is generally inefficient. In agreement with this notion, PMDs have also been found to be hypomethylated in cultured normal cell lines [21], [26], [27].

In Dnmt3a-deficient tumors, the methylome became globally hypomethylated. Dnmt3a-mediated protection against global hypomethylation was enriched in large chromosomal domains that showed a complementary distribution to PMDs. These Dnmt3a-protected domains (DPDs) are characterized by a higher overall CpG density and also showed higher levels of H3K4me1, a mark for active regulatory elements. Because of their higher functional significance, these regions might require a more effective mechanism for maintenance methylation. A role for Dnmt3a in effective maintenance methylation has been suggested before [28]–[30], and our study provides detailed insight into this feature.

The effects of Dnmt3a-dependent DNA hypomethylation on gene expression appeared to be complex. While we did not find any robust Dnmt3a-dependent methylation changes at promoter regions, we observed a strong enrichment of Dnmt3a-dependent hypomethylation in gene bodies. Interestingly, we found that the hypomethylation of relatively highly methylated 5′-UTRs was associated with higher transcript levels, which might reflect the close proximity of 5′-UTRs to gene promoters, where a silencing function of DNA methylation is well established [31]. We also found that hypomethylation of relatively highly methylated exons was associated with reduced transcript levels, which expands earlier findings [18]. The mechanistic role of exon methylation in gene regulation remains to be analyzed in future studies. It is interesting to note that gene body methylation has been shown to be enriched at splice junctions [32] and to be associated with polymerase II pausing and gene splicing [33]. Altered gene body methylation might thus promote tumor progression through complex quantitative and qualitative alterations in gene expression patterns.

## Materials and Methods

### Ethics statement

All experimentation with mice was carried out in accordance with the United States Health Research Extension Act, Public Law 99–158, “Animals In Research”. Specifically, the use of vertebrate animal subjects as described in this manuscript has been reviewed and approved by the Massachusetts Institute of Technology's Committee on Animal Care on 11/3/2011, under protocol no. 1110-096-13. Adequate procedures were taken to avoid unnecessary discomfort, pain, stress or injury to the animals, such as using appropriate surgical anesthesia and post-trauma analgesia as well as heat lamps and cage padding.

### Sample preparation

Adeno-cre virus infection of experimental animals and tumor harvest were as described previously [18]. Tumor genomic DNA was prepared as equimolar pools from 4 independent tumors. Large tumors were defined as tumors with more than 4 mm in diameter and small tumors with less than 2 mm. Normal lung genomic DNA was prepared as equimolar pools from lungs of three uninfected mice carrying one copy of K-ras^LSL G12D^ allele and being homozygous, heterozygous and wt for the Dnmt3a 2lox/2lox allele, respectively.

### Sequencing

Library preparation was performed as described previously [34]. Paired-end sequencing was performed on an Illumina HiSeq system with read lengths of 105 base pairs and an average insert size of 200 bp. Sequencing data have been deposited in the NCBI GEO database (accession number GSE38651).

### Methylation calling

Reads were trimmed to a maximal length of 80 bp and stretches of bases having a quality score <30 at the ends of the reads were removed. Reads were mapped using BSMAP 2.02 [35]. As a reference sequence for the bisulfite mapping we used the current assembly of the mouse genome (mm9). Only reads mapping uniquely and with both partners of the read pairs having the correct distance were used. Conversion rates were calculated for each library by determining the number of cytosines called in all mapped reads at all non-CpG positions and dividing it by the number of all bases in all mapped reads at all non-CpG positions. This ratio was subtracted from 1 to calculate the conversion rate. Methylation ratios were determined using a Python script (methratio.py) distributed together with the BSMAP package. Specifically, for both the forward and reverse strands all cytosine bases in the CG context in the reference sequence were identified independently. For all mapped reads in a given genomic interval, the number of cytosines in CG context that were called methylated as well as the number called unmethylated were counted.

### Methylation pattern analysis

For large-scale methylation analyses, a sliding window was iteratively shifted along each chromosome in a non-overlapping way. For each window position the average methylation ratio of all cytosines located in the respective window which were in CG context and which were covered by >0 reads was computed. Cumulated methylation ratios were used as an estimation for the methylation density in the respective window. Additional data were visualized using the UCSC Genome Browser (http://genome.ucsc.edu) using previously published results for nuclear lamina-associated domains [24], H3K4me1 distribution in normal mouse lung [36], gene expression in normal mouse lung [37] and gene expression in Dnmt3a^wt^ and Dnmt3a^KO^ tumors [18].

### Statistical analysis

The correlation of the different entities (methylation density, H3K4me1 density) with methylation differences between control samples and the Dnmt3a^wt^ tumors (PMDs) was determined by computing Spearman's rank correlation coefficients. The P-values were computed for the alternative hypothesis that the rank correlation coefficient is not equal to 0. All statistical computations were carried out using the statistical computing language R (version 2.14.1). Gene ontology analysis of differentially expressed genes was performed by using DAVID [38].

## Supporting Information

Figure S1 Mutational status of K-ras and Dnmt3a in various tissue samples. Local sequence coverage for K-ras (A) and Dnmt3a (B) is shown in green, red lines represent the smoothened sequence coverage. Orange shades indicate the gene targeting sequence. Prior to adenovirus-mediated expression of Cre recombinase, all mice had a K-ras^+/LSL-G12D^; Dnmt3a^2Lox/2Lox^ genotype. Cre-mediated recombination removes the LSL cassette from the mutant K-ras allele, thus activating the constitutively active G12D variant. In addition, Cre-mediated recombination removes exons 17, 18 and 19 from the mutant Dnmt3a allele, thus generating a catalytically inactive Dnmt3a variant.
(TIF)

Figure S2 Distribution of non-CpG methylation in various tissue samples. Histograms show the distribution of non-CpG methylation in several tissue samples, as indicated. Average methylation levels were determined for all covered non-CpG dinucleotides and then distributed into bins with increasing methylation ratios. White bars indicate unmethylated non-CpG dinucleotides, grey bars partially methylated non-CpG dinucleotides and black bars completely methylated non-CpG dinucleotides. White bars are cut off at 0.1, with actual ratios of 0.95 (control lung), 0.99 (3a^wt^ tumors) and 0.98 (3a^KO^ tumors).
(TIF)

Figure S3 Analysis of large-scale DNA methylation changes. Density plots of average DNA ratios for 20-kb windows covering the entire mouse genome. Dashed lines indicate a methylation loss of >0.15.
(TIF)

Table S1 Sequences used for the methylation analysis of repeats.
(DOC)

Table S2 Numbers of hypomethylated windows (100 kb).
(DOC)

Table S3 Gene enrichment analysis of differentially expressed genes.
(DOC)
