# Supplementary figures and images for: Dnmt3a Protects Active Chromosome Domains against Cancer-Associated Hypomethylation
Source: PLoS Genet. 2012 Dec 20;8(12):e1003146. doi: 10.1371/journal.pgen.1003146 (PMC3527206; doi:10.1371/journal.pgen.1003146)

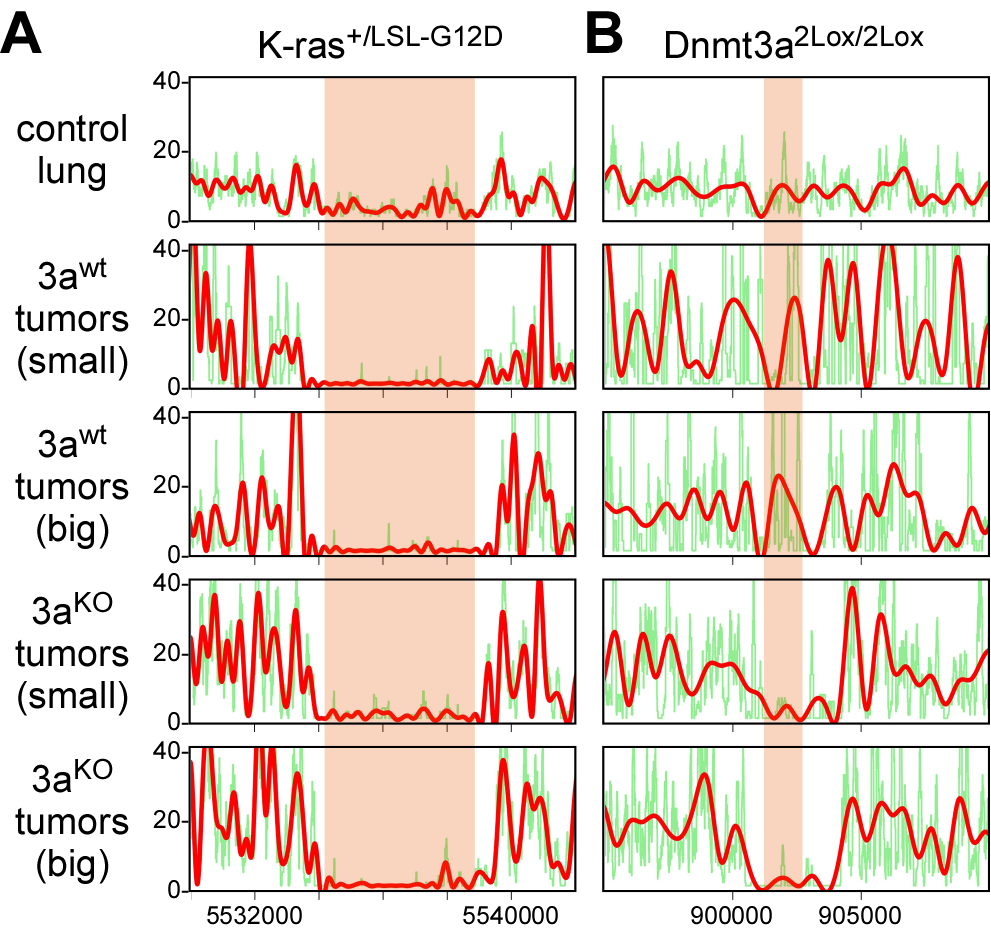

Supplement: Figure S1 — Mutational status of K-ras and Dnmt3a in various tissue samples. Local sequence coverage for K-ras (A) and Dnmt3a (B) is shown in green, red lines represent the smoothened sequence coverage. Orange shades indicate the gene targeting sequence. Prior to adenovirus-mediated expression of Cre recombinase, all mice had a K-ras+/LSL-G12D; Dnmt3a2Lox/2Lox genotype. Cre-mediated recombination removes the LSL cassette from the mutant K-ras allele, thus activating the constitutively active G12D variant. In addition, Cre-mediated recombination removes exons 17, 18 and 19 from the mutant Dnmt3a allele, thus generating a catalytically inactive Dnmt3a variant. (TIF) [file pgen.1003146.s001.tif]

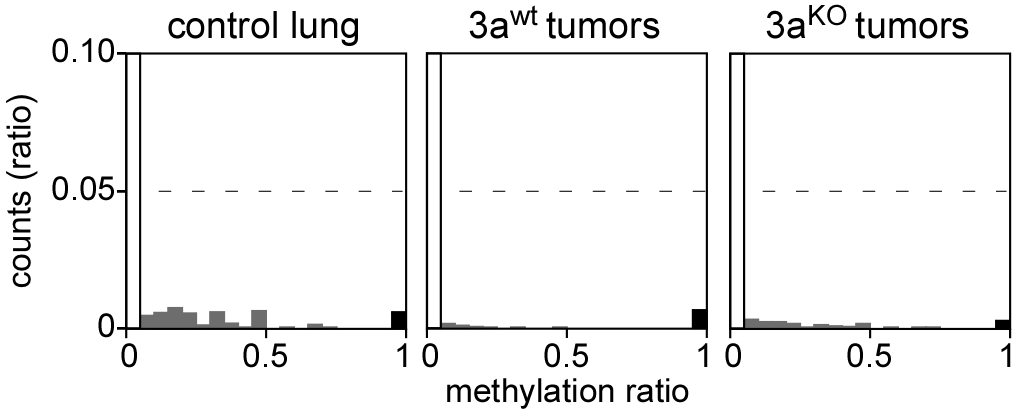

Supplement: Figure S2 — Distribution of non-CpG methylation in various tissue samples. Histograms show the distribution of non-CpG methylation in several tissue samples, as indicated. Average methylation levels were determined for all covered non-CpG dinucleotides and then distributed into bins with increasing methylation ratios. White bars indicate unmethylated non-CpG dinucleotides, grey bars partially methylated non-CpG dinucleotides and black bars completely methylated non-CpG dinucleotides. White bars are cut off at 0.1, with actual ratios of 0.95 (control lung), 0.99 (3awt tumors) and 0.98 (3aKO tumors). (TIF) [file pgen.1003146.s002.tif]

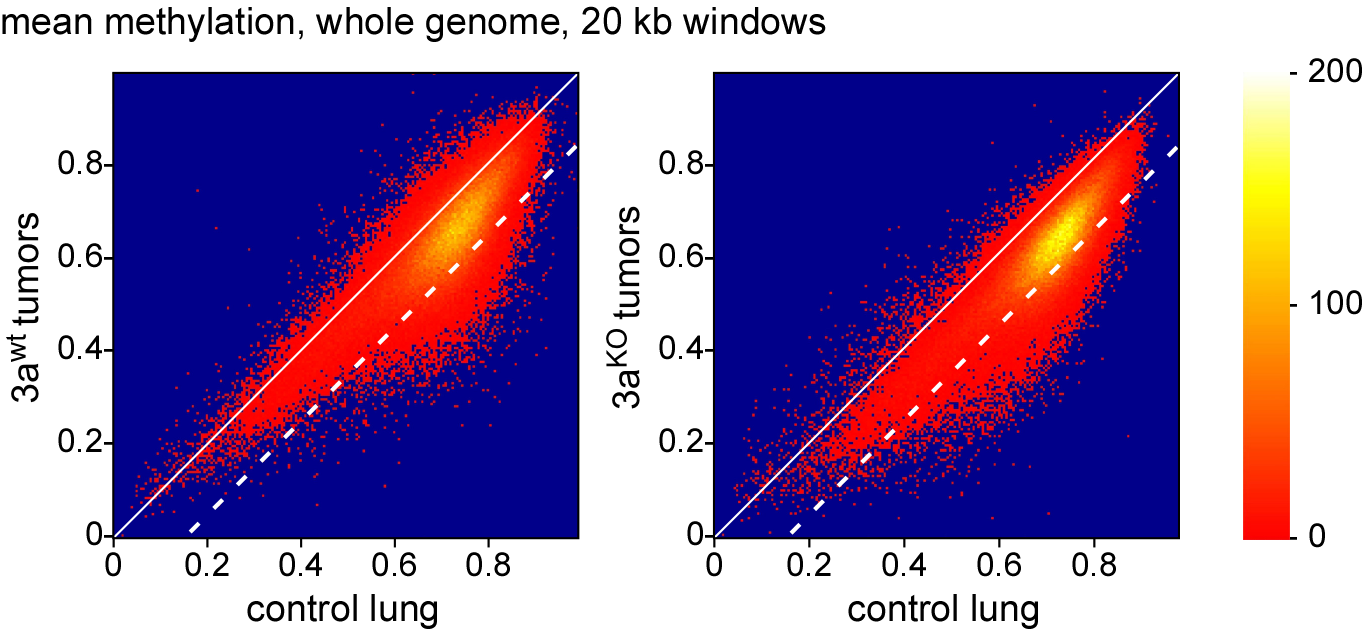

Supplement: Figure S3 — Analysis of large-scale DNA methylation changes. Density plots of average DNA ratios for 20-kb windows covering the entire mouse genome. Dashed lines indicate a methylation loss of >0.15. (TIF) [file pgen.1003146.s003.tif]
