# Supplementary material for: Dnmt3a Protects Active Chromosome Domains against Cancer-Associated Hypomethylation
Source: PLoS Genet. 2012 Dec 20;8(12):e1003146. doi: 10.1371/journal.pgen.1003146 (PMC3527206; doi:10.1371/journal.pgen.1003146)
Supplement: Table S1 — Sequences used for the methylation analysis of repeats. (DOC) [file pgen.1003146.s004.doc]

Table S1. Sequences used for the methylation analysis of repeats.

| **repeat** | **no. of copies** | **no. of CpGs** | **% of genome** |
| --- | --- | --- | --- |
| Line1 | 965,731 | 3,031,460 | 7.0 |
| SineB1 | 341,706 | 605,644 | 1.4 |
| sat | 8,020 | 17,888 | <0.1 |
