# Supplementary material for: Dnmt3a Protects Active Chromosome Domains against Cancer-Associated Hypomethylation
Source: PLoS Genet. 2012 Dec 20;8(12):e1003146. doi: 10.1371/journal.pgen.1003146 (PMC3527206; doi:10.1371/journal.pgen.1003146)
Supplement: Table S2 — Numbers of hypomethylated windows (100 kb). (DOC) [file pgen.1003146.s005.doc]

Table S2. Numbers of hypomethylated windows (100 kb).

| **hypomethylation** | **3a^wt^ vs. control** | **3a^KO^ vs. control** | **3a^KO^ vs. 3a^wt^** |
| --- | --- | --- | --- |
| >0.10 | 1316 | 12168 | 6751 |
| >0.15 | 100 | 2383 | 2154 |
| >0.20 | 11 | 345 | 329 |
| >0.25 | 1 | 58 | 26 |
