# Supplementary material for: Dnmt3a Protects Active Chromosome Domains against Cancer-Associated Hypomethylation
Source: PLoS Genet. 2012 Dec 20;8(12):e1003146. doi: 10.1371/journal.pgen.1003146 (PMC3527206; doi:10.1371/journal.pgen.1003146)
Supplement: Table S3 — Gene enrichment analysis of differentially expressed genes. (DOC) [file pgen.1003146.s006.doc]

Table S3. Gene enrichment analysis of differentially expressed genes.

| **Upregulated genes >2-fold)** | | |
| --- | --- | --- |
|  | | |
| **Cluster 1. Enrichment Score: 5.67** | | |
| Term | Count | P Value |
| secreted | 36 | 1.71E-07 |
| signal | 66 | 1.44E-05 |
| signal peptide | 66 | 1.35E-03 |
|  | | |
| **Cluster 2. Enrichment Score: 2.50** | | |
| Term | Count | P Value |
| cell adhesion | 15 | 2.72E-12 |
| biological adhesion | 15 | 2.77E-12 |
| homophilic cell adhesion | 6 | 3.14E-03 |
|  | | |
| **Cluster 3. Enrichment Score: 2.50** | | |
| Term | Count | P Value |
| acute-phase response | 5 | 1.13E-12 |
| response to wounding | 11 | 7.18E-11 |
| inflammatory response | 8 | 3.00E-03 |
|  | | |
| **Cluster 4. Enrichment Score: 2.12** | | |
| Term | Count | P Value |
| regulation of body fluid levels | 7 | 1.06E-12 |
| response to wounding | 11 | 7.18E-11 |
| coagulation | 5 | 2.90E-03 |

| **Downregulated genes (>2-fold)** | | |
| --- | --- | --- |
|  | | |
| **Cluster 1. Enrichment Score: 5.86** | | |
| Term | Count | P Value |
| glycoprotein | 80 | 8.89E-05 |
| signal | 70 | 1.31E-08 |
| disulfide bond | 59 | 2.10E-08 |
|  | | |
| **Cluster 2. Enrichment Score: 3.12** | | |
| Term | Count | P Value |
| lipoprotein | 20 | 9.15E-10 |
| gpi-anchor | 9 | 1.25E-12 |
| propeptide | 12 | 1.64E-11 |
|  | | |
| **Cluster 3. Enrichment Score: 2.90** | | |
| Term | Count | P Value |
| heme | 12 | 1.15E-10 |
| metalloprotein | 8 | 4.54E-10 |
| chromoprotein | 7 | 7.55E-09 |
|  | | |
| **Cluster 4. Enrichment Score: 2.34** | | |
| Term | Count | P Value |
| extracellular region part | 23 | 3.77E-12 |
| extracellular matrix | 9 | 4.23E-03 |
| proteinaceous extracell. matrix | 10 | 1.47E-02 |

Results were obtained by the functional annotation tool in the DAVID database using default thresholds. Shown are the top 3 annotation terms for all clusters with an enrichment score >2.
